# An Update on Flow Cytometry Analysis of Hematological Malignancies: Focus on Standardization

**DOI:** 10.3390/cancers17122045

**Published:** 2025-06-19

**Authors:** Eda Holl, Michael Kapinsky, Anis Larbi

**Affiliations:** 1 Beckman Coulter Life Sciences, Miami, FL 33196, USA; eholl@beckman.com (E.H.); mkapinsky@beckman.com (M.K.); 2 Department of Surgery, Duke University Medical Center, Durham, NC 27710, USA; 3 Department of Medicine, Faculty of Medicine and Health Sciences, University of Sherbrooke, Sherbrooke, QC J1N 3C6, Canada

**Keywords:** flow cytometry, leukemia, lymphoma, minimal residual disease, standardization, validated antibody panels

## Abstract

Flow cytometry use has significantly increased in clinical laboratories and has significantly helped improve the diagnosis of leukemias, lymphomas, and follow-up of minimal residual disease. Mastering this technique enables the performance of multiparametric single-cell analysis and increases the odds of identifying abnormal populations. As in many fields, there is a need to improve the quality of the data generated for accuracy, reproducibility, and trueness. The implementation of solutions reducing variability is achievable and needed, as the flow cytometry workflow involves many manual steps and items susceptible to operator bias and human error. Standardization of flow cytometry assays is sought and already implemented in many clinical hematology laboratories. However, the clinical community would highly benefit from further efforts in that direction to increase the comparability of findings across laboratories. This review covers the strengths and weaknesses of flow cytometry and focuses on the standardization approaches developed, including recent advances in the field.

## Simple Summary

Flow cytometry has significantly contributed to advances in the understanding and diagnosis of immunological and hematological conditions. We are reviewing in this article the basics of flow cytometry and how key steps in the flow cytometry experimental workflow could be affected by variability, introduced by the operator and/or by reagents/devices. We are focusing here on the role of flow cytometry in the diagnosis of hematological malignancies. We highlight (i) how standardization can improve the quality of the diagnosis in clinical laboratories and across multiple sites and (ii) ways to reduce the variability during flow cytometry analysis. The effort of several working groups is presenting aiming at defining better standards for the use of flow cytometry in clinical laboratories.

## 1. Principles of Flow Cytometry

**Figure 1:**
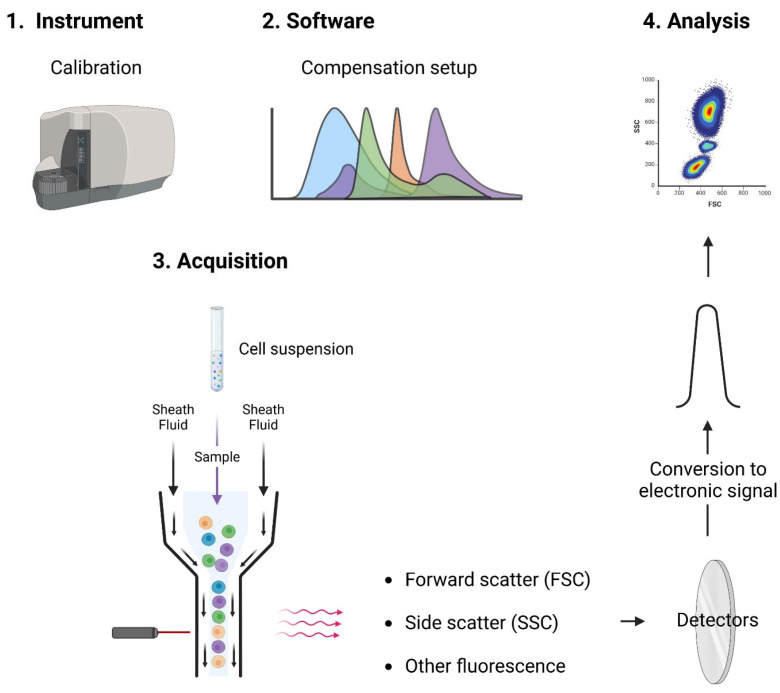
Principles of flow cytometry. Instrument setups include calibration, setting of voltages/gains with the unstained control samples, and determination of the compensation values. Acquisition of the sample after staining is enabled via hydrodynamic focusing aided by the sheath fluid that enables the alignment of cells into a single stream. The excitation of the individual cells by lasers induces the fluorescence emission that is detected and converted into an electronic signal for further analysis. The main components of the flow cytometer are the fluidic, optical, and electronic systems.

Flow cytometry has revolutionized the field of hematology [1,2,3], offering a powerful tool for the diagnosis, classification, and monitoring of hematological malignancies [4]. This chapter delves into the critical role of flow cytometry in understanding and diagnosing these complex blood cancers, highlighting its principles, applications, and the advantages it brings to the clinical setting. Flow cytometry is a technology that allows the rapid analysis of multiple physical and chemical characteristics of single cells or particles [5] as they flow in a fluid stream through a beam of light, typically a laser (Figure 1). Key parameters measured include cell size (forward scatter or FSC), granularity (side scatter or SSC), and the expression of specific cell surface and intracellular markers. By labeling cells with fluorescent antibodies targeting these markers, flow cytometry can provide detailed information about the phenotype and function of individual cells within a heterogeneous population [6,7,8]. Optical filters and mirrors direct this emitted light to the corresponding detectors. The detection system captures and converts the light signals into electronic signals that can be analyzed. Software programs collect, process, and display the data in a format that can be easily interpreted by the user.

The flow cytometry process involves several steps [9]:

• Cytometer preparation: Experiments require calibration and optimization of cytometer setup (such as compensation) to simplify subsequent analysis.
• Sample preparation: Dedicated protocols are used (e.g., red blood cell lysis) and cells must be in a single-cell suspension, free from clumps and debris.
• Sample staining: Cells are stained with fluorescently labeled antibodies or dyes that bind to specific cellular components, such as surface markers, intracellular proteins, or DNA.

The acquired data are analyzed to interpret the results [10]:

• Gating: The process of selecting specific cell populations based on their scatter and fluorescence characteristics. Gating is crucial for identifying and analyzing subpopulations of interest.
• Histograms and Dot Plots: Data are displayed in various formats, such as histograms (single-parameter plots) and dot plots (dual-parameter plots), to visualize the distribution and relationships of different cell populations.
• Quantitative analysis: The software allows for the semi-quantitative (units are mean fluorescence intensities) assessment of marker expression, cell counts, and other parameters.

One of the primary applications of flow cytometry in hematology is immunophenotyping, which involves the identification of cell surface and intracellular proteins to classify cells [11]. This is particularly important in diagnosing different types of leukemia and lymphoma [12]. The rise in cytometry started with its use in the diagnosis of leukemia [13], and with the increased availability of fluorescence-conjugated antibodies, it has significantly evolved [14].

Flow cytometry offers several advantages that make it an invaluable tool in the diagnosis and management of hematological malignancies:

• Speed and efficiency (high throughput): Analyze thousands of cells per second, providing rapid results that are essential for timely diagnosis and treatment decisions such as for acute leukemias where prompt treatment initiation is critical.
• Multiparametric analysis at the single-cell level: Simultaneously measure multiple parameters on individual cells for a comprehensive analysis of populations, enhanced accuracy of diagnosis (distinguish different types of malignancies).
• Quantitative assessment: Provide semi-quantitative data on the expression levels of markers, which can be useful for monitoring disease progression and response to therapy (minimal residual disease (MRD)). Quantitative mean fluorescence intensities (MFIs) can be obtained by calibration against defined binding sites.
• Versatility: This can be applied to a wide range of sample types, including peripheral blood, bone marrow, and lymph node biopsies.

## 2. Importance of Flow Cytometry for Hematological Malignancy Diagnosis

### 2.1. Low Complexity Analysis

Flow cytometry has the advantage of providing high-throughput single information which enables the identification of aberrant expression profiles and enables deep classification of the type and subtype of hematological abnormalities when the normal phenotype is well established. However, it does not always require numerous markers to show an abnormal profile in a biological sample. A simple DNA content analysis enables us to identify ploidies in a cell population, e.g., to classify B-ALL [15]. While blasts usually show low scatter (SSC) and CD45 expression, other cells, may also be dim to intermediate in this gate (basophils, dendritic cells, hematogones), the addition of additional maturation markers (eg. CD11b, CD15) or immature cells (eg. CD34, CD117) becomes necessary. The clonality of mature B-cells can be assessed with the immunoglobulin light chain expression (kappa and lambda). Neoplastic B-cells express only one class of light chain (kappa or lambda), while normal B-cells always display a mixed profile. The clonality of mature T-cells is assessed by measuring the TCR Vβ repertoire or even more simply with constant TCRβ chain 1 and 2 expressions (TRBC1 and TRBC2). The simple measure of TRBC1/TRBC2 could provide a simpler workflow for the identification of T-cell neoplasms [16].

### 2.2. Higher Complexity Analysis

Together with histology, morphological, and other information, multiparametric flow cytometry enables rapid diagnosis and classification of leukemia/lymphoma. Nowadays, a series of markers are used for the identification and characterization of leukemias and lymphomas, and this has been developed by several working groups. The following list of markers (Table 1) utilized is not an opinion nor a recommendation but a summary of what has been developed and agreed upon by several groups [17,18,19,20,21,22,23] based on the fifth edition of the World Health Organization (WHO) classification of haematolymphoid tumors [24] and the Bethesda guidelines [25]. Utilizing standardized panels and protocols can ensure consistency and accuracy in disease monitoring across different laboratories [26].

### 2.3. The Limitations and Complementarity of Flow Cytometry

Diagnosing suspected blood neoplasms involves a suite of laboratory techniques. It often starts with a CBC, and if abnormal, a microscopic examination of blood or bone marrow smears is performed. Microscopy reveals morphological details but requires expertise and does not definitively identify cell lineage. Other crucial methods provide more in-depth information, including bone marrow biopsy, histology, immunohistochemistry, cytogenetic analysis, and molecular testing [27]. Flow cytometry is a vital complementary technique. It is faster than immunohistochemistry and allows for simultaneous analysis of multiple characteristics on single cells, though it needs fresh, viable samples and does not show cell morphology. Flow cytometry requires careful sample preparation as indicated for the assessment of blast counts [28]. Cytogenetic and molecular testing is essential for classifying and staging acute leukemias; flow cytometry alone is not sufficient for this. Flow cytometry has not been useful for diagnosing Hodgkin lymphoma but recent development has improved this [29]. Traditional flow cytometry for T-cell clonality (TCR Vβ) is complex, labor-intensive, and less sensitive, so it is not commonly used in laboratories [30]. For maturing myeloid neoplasms, diagnosis primarily relies on clinical data, morphology, and genetic testing; flow cytometry-based diagnosis value is limited despite often finding abnormal cell phenotypes. For minimal residual disease detection, RT-qPCR for IG/TCR gene rearrangements is a common method, but it is time-consuming because it requires developing a specific test for each patient and needs sufficient original sample DNA [31]. It also cannot detect new subclones. dPCR is often more accurate than RT-qPCR, with higher amplification efficiency, less affected by inhibitors, and provides absolute quantification. It is faster than NGS and easier to analyze but requires validation and specific assay design [32]. Flow cytometry assays are more rapid than molecular techniques but require analysis of a high number of cells. Despite some limitations, flow cytometry remains a crucial complementary tool in the diagnosis of hematological malignancies. While methods like morphological examination of blood and bone marrow provide vital structural details and preliminary clues, and techniques such as cytogenetics and molecular testing are essential for precise classification and risk stratification based on genetic alterations, flow cytometry excels at rapidly and quantitatively identifying specific cell populations based on their surface and intracellular protein expression.

## 3. Pitfalls in Flow Cytometry Analysis

### 3.1. Overview

Recent advancements in flow cytometry (sensitivity, software, and reagents) have improved its utility for the diagnosis and monitoring of diseases; however, combining >8 antibodies in a single tube can introduce technical and interpretative challenges that are complex. While multiparameter panels facilitate the identification of abnormal cell populations, the characteristics of neoplastic populations can create potential diagnostic pitfalls [33]. A thorough understanding of normal phenotypic patterns in states of rest, recovery, and activation is essential for accurately identifying the abnormal populations characteristic of hematopoietic neoplasms [34,35]. Additionally, incorporating newer therapeutic strategies, particularly targeted therapies, can complicate standard methods for flow cytometric data analysis [36]. While flow cytometry is a powerful diagnostic tool, there are challenges and considerations to keep in mind:

• Expertise: The interpretation of flow cytometry data requires specialized training and expertise. Skilled cytometrists are essential for accurate diagnosis, as biology and aberrant expression are not always predictable [37,38]. Practitioners must have a deep understanding of the whole process from panel design, sample preparation, instrument calibration, and quality control to complex data interpretation. This involves understanding the normal/expected expression of specific antigens during the development/maturation of hematopoietic cells compared to aberrant/unexpected expression in the case of leukemia/lymphoma populations [39,40,41,42].
• Standardization: Inadequate laboratory performance can have far-reaching consequences for practical medicine, the healthcare system, and ultimately for the patient. Poor-quality results may lead to incorrect interpretations by clinicians, potentially worsening the patient’s condition [43]. Hence, standardization of methods and quality control measures are important to ensure consistency and reliability [44]. Implementing standardization is not necessarily an easy process [43,45] but could be achieved via (i) establishing a reference system that includes reference methods and materials, (ii) calibrating measurement procedures using the established reference system, (iii) verifying the comparability of measurements used in patient care, typically by measuring a set of authentic patient samples to ensure uniformity of results across different methods, and (iv) simplifying workflows, reducing operator intervention to remove potential bias and to set the stage for automation.
• Cost and Accessibility: Efforts to reduce costs and improve access are important for broader implementation [46,47]. Of note, implementing flow cytometry screening for patient monitoring such as in the case of MRD could reduce the overall healthcare cost [48]. Hence, a critical analysis of flow cytometry-based assay costs should be performed with a holistic view of costs for the initial diagnostic, treatment, and follow-up phases. The number of markers used for diagnosis can be limited by costs for the patients or the reimbursement policy, which is highly variable depending on the country or region [49].

### 3.2. Validation of Flow Cytometry Assays

Flow cytometry clinical laboratories either develop their own assays (LDTs: lab-developed tests) or utilize in vitro diagnostic (IVD) assays. Prior to commercialization, IVD assays are validated and submitted to regulatory bodies and laboratories need simply to verify they can reproduce the performance of the assay in their laboratory. On the other hand, LDTs require an extensive validation of the assay performance and other laboratories willing to utilize the LDT need to perform their own validation. Each component of the assay should be included in the validation plan. This should include the method, the reagents, equipment, and supplies used, the number and type of samples used in each validation experiment, the statistical analysis performed on each experiment as well as acceptance criteria for each parameter. The performance specifications included in validation are accuracy (or trueness), precision (reproducibility and repeatability), detection capability, selectivity, reference range, and sample/reagent stability. Accuracy is defined as the closeness of agreement between the average values obtained from a large series of test results when compared to an accepted reference standard. Precision is defined as the dispersion of replicate measurements when a variable is introduced (operator, runs, instruments). Selectivity is often defined before the validation step by careful selection of antibody clones and washing steps required.

## 4. A Deeper Look into Flow Cytometry Standardization

### 4.1. The Need for Standardization

The standardization of assays in clinical laboratories has improved the quality of diagnosis and overall care of patients. In flow cytometry, several types of issues arise when a new assay is developed. Typical pre-analytical issues in flow cytometry are signs of poor handling after collection such as clotted or hemolyzed samples [50]. Optimal RBC lysis (erythrocytes < 10%) was recently shown to be achieved using NH_4_Cl or VersaLyse®, while lyse-no-wash protocols have been tested and validated [51]. Similarly, certain samples may show resistance to red blood cell lysis due to the disease state or therapy such as in AIDS patients on therapy or patients with liver diseases [52]. Among the fluorescent dyes developed are tandem dyes, and their decoupling should be avoided. This is an important aspect to take into consideration when designing the panel [53]. This can be avoided when dry formulations of premixed antibody cocktails are used while liquid premixes show significant tandem dye oxidation.

Analytical issues also exist and relate to the state and stability of the cytometer being used. This includes laser alignment and the choice of the appropriate setting (voltage/gain) for optimal signal-to-noise ratio. Defining the optimal compensation to mitigate spectral overlap is an essential piece of the experimental setup [54]. Elimination of doublets and selection of the events before/after an interruption of the recording can help remove unwanted events. When performing minimal residual disease monitoring, it is essential to ensure the cleanliness/stability of the cytometer, as a very low number of cells are searched.

**Figure 2:**
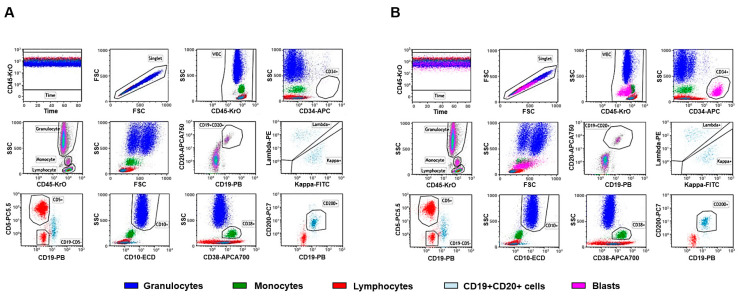
Control samples for hematological cases. (**A**) Representative dot plots of normal control cells for B-cell neoplasm. (**B**) Representative dot plots of abnormal control cells for B-cell neoplasm. Stabilized control cells were stained with CD45, CD19, kappa, lambda, CD10, CD5, CD200, CD34, CD38 and CD20 from the ClearLLab 10C Panels (B-cell tube shown here).

The post-analytical phase heavily relies on expertise in hematological malignancies [55]. Neoplastic populations may mimic a state of maturation or activation but will exhibit aberrant phenotypic patterns that are absent in healthy individuals. These abnormalities can manifest as increased, decreased, or absent antigen expression relative to their normal counterparts, collectively termed as ‘different from normal” [56]. The foundation for interpreting flow cytometric data in the identification of hematopoietic neoplasms, therefore, relies heavily on a comprehensive understanding of normal immunophenotypic patterns [57]. Aberrant phenotypes may also escape from the expected gating strategy and additional markers are useful in order not to miss the identification of such cells [58]. Control samples (Figure 2) aid in (i) verifying the antibody cocktail, (ii) checking reagents do not exhibit degradation, (iii) ensuring equipment calibration, and (iv) ensuring the gating strategy and analysis template are correct [59].

### 4.2. Standardization and Harmonization

The WHO provides comprehensive guidelines for the analysis, reporting, and management of hematological malignancies. These recommendations aim at ensuring accurate diagnosis, classification, and monitoring of blood cancers. Several groups were involved in the standardization of flow cytometry experiments for the diagnosis of hematological malignancies [60,61]. This has become a necessity to counter the drawbacks regarding confidence in and comparability of this technology. The Clinical and Laboratory Standards Institute guideline H62 outlines best practices for flow cytometry measurements, emphasizing quality management, quality assurance (QA), and quality control (QC). This guideline was developed collaboratively by industry representatives, government agencies such as the US FDA, the National Institute of Standards and Technology, and professional experts [62]. The primary objectives of QA and QC are to ensure that instruments and assays consistently operate in a qualified state, that new reagent lots are comparable through testing, and that staff maintain ongoing competency in assay performance [63]. Due to the variety of reagents used in flow cytometry assays, monitoring reagent continuity is a vital component of QA and QC.

Some of the recommendations from the various working groups are summarized here:

• Reagent and Instrument Standardization: Use standardized reagents, protocols, and calibration of flow cytometers to ensure consistent and reproducible results.
• Quality Control: Implement robust quality control measures to monitor instrument performance and reagent quality regularly.
• Comprehensive Marker Panels: Design antibody panels that cover a broad range of lineage-specific and differentiation markers to accurately identify and classify hematological malignancies.
• Disease-Specific Panels: Customize panels based on suspected malignancy, such as acute leukemia, chronic leukemia, or lymphoma.
• Sample Preparation: Ensure proper handling and preparation of samples to maintain cell integrity and viability and prepare single-cell suspensions to avoid clumping and ensure accurate analysis.
• Data Acquisition: Acquire high-quality data using appropriate laser settings and compensation controls, ensuring proper instrument alignment, and employing rigorous gating strategies to accurately identify and analyze subpopulations of interest.
• Data Analysis and Interpretation: Data should be analyzed by experienced personnel who are proficient in flow cytometry techniques and the interpretation of hematological malignancies and utilize advanced software tools for comprehensive analysis, including the identification of MRD.
• Clear Reporting: Provide clear and detailed reports that include information on the markers used, gating strategies, and interpretation of results.
• Clinical Correlation: Correlate flow cytometry findings with clinical features and other laboratory results for accurate diagnosis and management.
• Continuous Education and Training: Ensure continuous education and training for laboratory personnel to stay updated with the latest advancements and best practices in flow cytometry.

**Figure 3:**
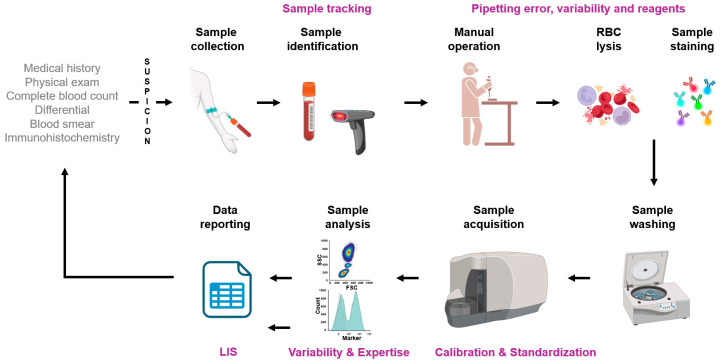
Flow cytometry workflow in the clinical laboratory. The scenario proposed here depicts a workflow highly dependent on human intervention. In purple are the steps where gaps exist and may benefit from standardization.

While reproducibility within the same laboratory can be maintained at high standards this becomes more difficult when an assay is transferred to another laboratory. The community has benefited a lot from the guidelines of the International Council for Standardization of Haematology (ICSH) and the International Clinical Cytometry Society (ICCS) that describe the rationale for the validation of assays, the avoidance of pre-analytical, analytical, and post-analytical issues, and assay performance criteria [64,65]. Standardization involves a comprehensive and detailed series of interconnected methodical steps, supported by standard operating protocols (SOPs), designed to eliminate any recognized sources of variation. This includes pre-analytical steps (such as sample preparation and immunostaining), analytical steps (like instrument setup), and post-analytical steps (including analysis and interpretation) (Figure 3). In contrast, harmonization refers to an approach where only the key steps of the entire process are performed in a similar manner, while other parts are executed using locally preferred methods. Typically, this can refer to harmonizing the set of markers to apply when testing for a specific condition, and which materials to interrogate.

Generally, while standardization can achieve all the reproducibility goals, harmonization can still ensure reproducible interpretation and enumeration. These terms represent a continuum that can evolve through interlaboratory studies. Harmonization can serve as a practical and attainable initial step, which may gradually progress into a more comprehensive set of methods that constitute standardization [66]. Several studies detailed below are shown as examples of standardization and/or harmonization of flow cytometry studies in the context of hematological malignancies [67,68,69].

### 4.3. Implementation of Standardization

Several studies have shown the feasibility and benefits of standardization in multicenter studies [70,71]. A study has reported the standardization of CD30 measurement in non-Hodgkin lymphoma by flow cytometry. The results indicate that flow cytometry agreed with immunohistochemistry (IHC) in 77% of cases. Some of the discrepancies could be attributed to differences in clones. Notably, flow cytometry was more sensitive than IHC in 11% of cases, particularly in cases of diffuse large B-cell lymphomas. This was performed in the context of Brentuximab Vedotin (BV) treatment for lymphoma, a CD30 monoclonal antibody (Ab)-drug conjugate, where the response is not always correlated to CD30 expression detected by immunohistochemistry (IHC). Hence, implementing multicenter standardized flow cytometry for specific CD30 detection could enhance case identification and broaden the application of BV treatment to various CD30-positive lymphomas [72]. The group behind these findings has also proposed a standardization approach for the follow-up of MRD in acute lymphoblastic leukemia. Determination of MRD by flow cytometry consists of the identification of aberrant phenotypes such as cross-lineage antigen expression (myeloid antigens in ALL), asynchronous patterns of expression of maturation markers (Ig on CD34+ cells), and abnormal levels of expression of individual markers [73]. Some of the limitations in MRD assessment by flow cytometry include the following: (i) the low number of target cells in the sample to test, (ii) the alike phenotypes between healthy precursor cells and leukemia cells, (iii) the impact of treatment on the expression of targeted markers (glucocorticoid therapy on CD10, CD19, CD34 and others), and (iv) the lack of standardization. Hence, increasing the number of markers for the assessment of MRD in ALL, when possible, could be a solution to prevent some of these issues. A detailed description of the recommendations is provided here [74] and updated recently [75]. A similar approach was proposed for the detection of rare events including CD34+ counting, monitoring of Paroxysmal Nocturnal Hemoglobinuria, and monitoring of MRD [76].

The European Research Initiative on CLL (ERIC) and the European Society for Clinical Cell Analysis (ESCCA) have initiated a harmonization project to identify reproducible criteria and consensus on markers recommended for the diagnosis of CLL [77]. This was initiated as there is no gold standard for the diagnosis of CLL and some of the characteristics used (sIg and CD20) show some issues with reproducibility across different laboratories. The ERIC/ESCCA members classified markers as required or recommended for CLL diagnosis. More than 14,000 cases from 13 different centers were tested. The consensus for required diagnostic markers included the following: CD19, CD5, CD20, CD23, kappa, and lambda. The recommended additional markers potentially useful for differential diagnosis were as follows: CD43, CD79b, CD81, CD200, CD10, and ROR1. The British Society for Haematology also engaged in such standardization/recommendation guidelines which were published recently [15,16,17,18] and focused on mantle cell lymphomas and marginal zone lymphomas. In the case of mantle cell lymphoma, it is recommended to use CD19, CD20, CD79b, CD5, FMC7, CD200, CD10, CD23, and surface immunoglobulin that could be complemented with CD22 and ROR1. Other initiatives such as the EuroFlow consortium have proposed sets of markers and protocols preferentially used in the context of MRD for chronic lymphocytic leukemia and acute lymphoblastic leukemia, acute leukemia subtypes, or the analysis of bone marrow samples for acute myeloid leukemia [78,79,80,81]. This extensive effort can be curated here [82]. For some time, the antibody panels proposed by EuroFlow were unique as they underwent full technical and clinical validation across various sites and were used as a reference in many clinical laboratories. However, a significant number of these laboratories have now adapted the workflow/panels to their settings and sourced reagents from other vendors which has contributed to inconsistency across different settings.

## 5. Update on Solutions Improving Resolution and Reducing Variability

### 5.1. Ready-to-Use Reagents

The burden of validating laboratory-developed tests (LDTs) is both substantial and complex, involving a spectrum of regulatory, technical, and operational challenges [9]. Laboratories must ensure their assays meet exacting standards of accuracy, precision, specificity, and sensitivity. This necessitates comprehensive validation protocols, including stringent analytical and clinical performance evaluations, to confirm test reliability and robustness. Furthermore, staying up to date with evolving regulatory requirements and technological advancements adds layers of complexity and resource demands. The financial costs, time investment, and specialized expertise needed for LDT validation can be considerable, exerting significant pressure on laboratory personnel and resources. Additionally, the obligation to maintain thorough documentation and rigorous quality assurance measures intensifies the burden, ensuring that tests consistently yield reliable and reproducible results critical for patient care. Hence, the development of ready-to-use solutions significantly simplifies laboratory workflows. Such regulatory-approved solutions may combine reagent cocktailing and stabilization to extend their use in time. The stabilization process can be performed in various ways including lyophilization [83] and drying [84]. Early studies have reported the successful identification and phenotypic characterization of abnormal populations from specimens submitted for routine flow cytometry testing [85]. The ready-to-use dried cocktail mixes showed 100% agreement with LDTs regarding the absence of abnormal population, and 95.3% agreement with abnormal population identification. The disadvantage of such ready-to-use solutions is the lack of identification of abnormal populations when a critical marker is needed but absent in the antibody cocktail. The manual preparation of LDT panels can lead to errors and is time-consuming, posing significant challenges in their development, validation, and use. Hence, ready-to-use stabilized reagents should reduce laboratory indirect costs and improve efficiency and accuracy [86]. Such panels have been successfully utilized and implemented in the examination of different hematological malignancies [87,88,89]. This has shown to also be useful in the context of multicenter studies [85]. The study of 453 samples in North America and Europe showed that from 198 samples with no malignancy and 255 with an abnormal population the diagnostic accuracy of ready-to-use dried solutions has proven very good, with a sensitivity of 96% and specificity of 95% with 98% agreement with LDTs. The use of stabilized reagents has also been used in the context of immunological assessment of biological samples [66]. It has proven to reduce significantly the site-to-site variability as demonstrated in the ONE Study and the Human Immune Phenotyping Consortium [90,91]. The ONE Study validated flow cytometry panels for analysis of whole blood samples and has been used as a reference for a subsequent study which was a collection of seven investigator-led, single-arm trials carried out internationally at eight hospitals in France, Germany, Italy, the UK, and the USA [92]. As commented by the authors “the assay complexity in combination with a diversity of equipment, reagents, and many other pre-analytical factors such as specimen age, staining procedures, compensation, and analytical factors such as subset definition, also increases the variability, particularly when comparing results obtained within different laboratories …the consortium developed a robust immune monitoring procedure to profile peripheral blood cellular phenotype and function of whole blood (WB) leukocytes based on flow cytometry…the results of the performance evaluation of the panels described here demonstrate highly reproducible SOPs that are applicable in general for use in multicenter clinical trials.”.

**Figure 4:**
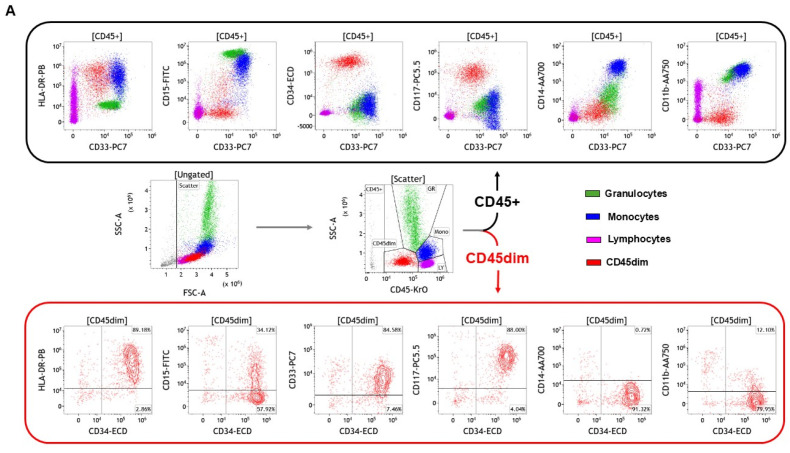

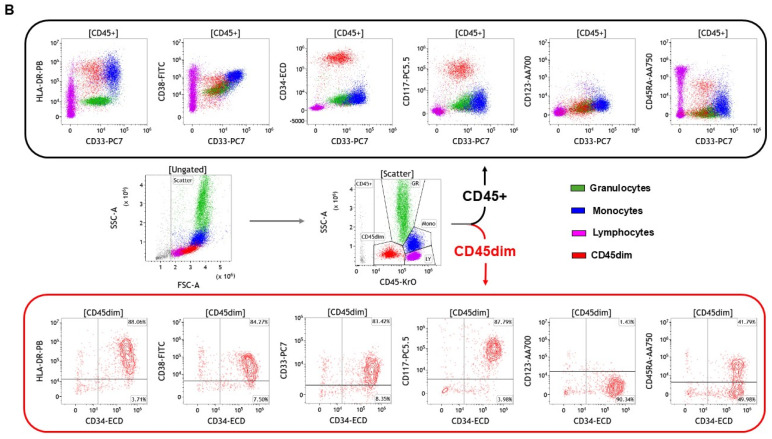
Residual cell analysis with flow cytometry. Samples were stained with ready-to-use stabilized cocktails for the assessment of abnormal marker expression and the maturation stage of myeloid progenitor cells. (**A**) Analysis of a blood sample using the Rare Events AML1* DURAClone tube. (**B**) Analysis of a blood sample using the Rare Events AML2* DURAClone tube (Beckman Coulter Life Sciences, Brea, CA, USA). * For research use only. Not for use in diagnostic procedures.

One challenge in the investigation of hematological malignancies is the ability to detect abnormal populations are samples with low cell counts. The same dried cocktails were proof-tested after serial dilutions of normal and abnormal samples (final cell concentrations from 3.0 × 10^6^ to 0.0469 × 10^6^ cells/mL). Across all patients, the antigen expression pattern remained consistent at all tested cell concentrations [93]. Furthermore, the mean deviation of the MFI from the value obtained using 3.0 × 10^6^ cells/mL did not exceed 10% for any of the antibodies. The tests conducted with the lowest cell density yielded identical patterns of antigen expression in all patients as those performed with the FDA-approved concentration, demonstrating 100% concordance between the two protocols. A more real-life example exists: cerebrospinal fluid (CSF) analysis [94]. A study showed the sensitivity of the flow cytometry method for the analysis of B-ALL in CSF using dried reagents [95]. In conclusion, the utilization of stabilized ready-to-use cocktails greatly simplifies laboratory workflows and sustains the desired sensitivity and specificity for the identification and characterization of hematological malignancies (Figure 4).

### 5.2. Minimal Residual Disease: The Reproducibility Perspective

The use of stabilized reagents described above brings reproducibility and ease of use to the next level. Applying this to rare events enables more accurate tracking of the number of residual cells in disease evolution. The fact the concentration of antibodies is strictly the same in a cocktail of dry reagents significantly increases the accuracy of the flow cytometry analysis during follow-up. Removing the error of manual pipetting by the operators is crucial. Enumeration and analysis of rare cells in the context of AML in blood or bone marrow is possible through carefully optimized and titrated antibody combinations. This has been carried out with the following markers: HLA-DR, CD33, CD34, CD45, CD117, CD15, CD14, and CD11b for the assessment of abnormal marker expression and with HLA-DR, CD33, CD34, CD45, CD117, CD38, CD123, and CD45RA for the assessment of the maturation stage of myeloid progenitor cells (Figure 4). Still, the development of panels targeting a wider variety of rare cells with CE-IVD and FDA regulatory approvals is necessary.

### 5.3. Minimal Residual Disease: The Sensitivity Perspective

Specificity and sensitivity are two important characteristics of diagnostic tests. Sensitivity is probably the most important aspect of minimal residual disease monitoring, as the aim is to detect very few cells within the pool of leukocytes [96]. A study has compared the advent of next-generation sequencing and flow cytometry for MRD in myeloma. Authors used NGS targeting the IGH and IGK genes for clonal characterization and CD117, CD19, CD138, CD56, CD45, CD38, and κ/λ antibodies for flow cytometry analysis [97]. The concordance between the two techniques was 92.9%, and discordant cases were all with low-level disease near detection limits for both assays. While both techniques show similar sensitivity, they highlight the need for more sensitive techniques for samples with very low-level disease.

**Figure 5:**
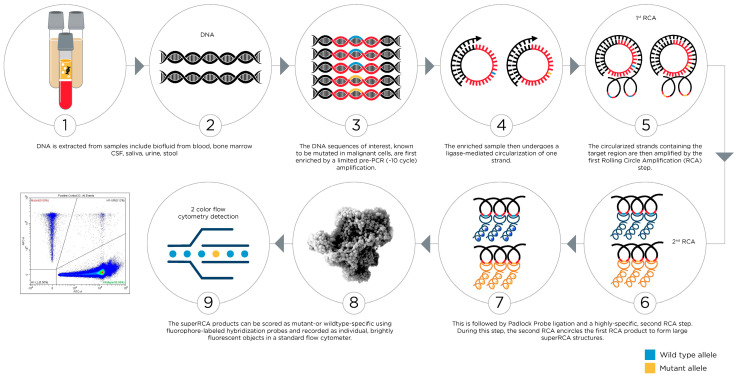
Generation of SuperRCA amplification products. DNA sequences of interest in a sample are first amplified using PCR. The amplified DNA strands are then converted into single-stranded DNA circles through templated ligation of their 5′ and 3′ ends. Oligonucleotides that facilitate the circularization reactions subsequently act as primers for Rolling Circle Amplification (RCA) reactions. The RCA products are then targeted with padlock probes specific to either mutant or wild-type sequences. Ligated padlock probes, wrapped around the RCA products, subsequently serve as templates for secondary RCA reactions, which are primed by an added oligonucleotide. For each initial DNA circle, the reaction produces large clusters of mainly single-stranded DNA objects, known as SuperRCA products. These SuperRCA products can bind up to a million fluorescence-labeled hybridization probes, enabling efficient counting through standard techniques such as flow cytometry. The sensitivity of the assay ranges from 10^−4^ to 10^−6^ compared to digital droplet PCR 10^−3^ to 10^−4^.

Recently, the SuperRCA® (Rolling Circle Amplification) assay, a highly sensitive and specific diagnostic tool used to detect minimal residual disease, was developed [98]. The SuperRCA® assay employs a unique amplification technique (Figure 5) that enhances the detection of rare cancer cells by amplifying specific DNA or RNA sequences associated with the malignancy [99]. This method improves the accuracy of rare cell detection compared to traditional techniques, potentially allowing for better monitoring of disease status and guiding treatment decisions to improve patient outcomes [100]. The SuperRCA® assay can detect 1 mutation out of 100,000 wild-type DNA molecules. With this high sensitivity, the SuperRCA® assay is a valuable tool in the management of cancer, helping to achieve more precise and personalized treatment strategies.

The SuperRCA® assay procedure is ideal for routine use due to its high sensitivity and simplicity. The 5 h protocol only requires five sequential additions to a DNA sample, each followed by incubations, before the reaction products are analyzed using a standard flow cytometer. More than 5 million such objects are generated in each reaction, providing excellent quantitative precision without the need for specialized equipment beyond a flow cytometer. While target sequences prone to mutation in tumors, such as GC-rich regions, can pose challenges for sequence distinction, the SuperRCA® procedure offers broad target compatibility. While a risk of mistyping due to polymerase error may exist, the use of a high-fidelity polymerase keeps this risk below the detection threshold. Rare cell detection through the SuperRCA® assay might prove to be a useful tool/biomarker assay in drug development as MRD assessment can be used as a surrogate marker which can expedite clinical trials and lead to approvals. It can also be used in patient selection/stratification in order to select patients at high risk or enrich the trial population. For instance, in ALL, MRD is one of the most significant prognostic factors, independent of patient age, B- or T-cell origin, or genetic subtype.

## 6. The Future of Flow Cytometry for Hematological Malignancies

### 6.1. Spectral Flow Cytometry

Spectral flow cytometry is an advanced technique used in the field of cytometry that allows the detection and analysis of more parameters simultaneously. Unlike traditional flow cytometry, which relies on discrete bandpass filters to detect signals from fluorochromes, spectral flow cytometry captures the entire emission spectrum of each fluorochrome across a wide range of wavelengths [101]. The technique developed in the early years of this century employs a prism or diffraction grating to disperse emitted light from fluorochromes across a detector array, typically a series of photomultiplier tubes (PMTs) or avalanche photodiodes (APDs). This allows for the measurement of a continuous spectrum for each cell. The collected spectral data are processed using sophisticated unmixing algorithms that deconvolute the overlapping emission spectra of different fluorochromes. This computational approach enables the accurate separation and quantification of signals from multiple fluorochromes, even those with closely overlapping spectra. Autofluorescence presents a significant challenge in the precise delineation of gates, particularly affecting certain myeloid and plasma cell populations [102]. Spectral flow cytometry offers the potential to mitigate these issues and improve gating accuracy, especially in the context of leukemic and plasma cell analysis [103,104]. Nevertheless, the clinical application of spectral flow cytometry remains limited, and it is not yet clear if these theoretical advantages will translate into enhanced diagnostic performance. Moreover, the laboratory workflow with spectral cytometry is more complex making it less suitable for clinical flow cytometry applications that require straightforward processes. Rigorous validation studies are required, and comparisons are made to conventional flow cytometers that have received regulatory approval. With regulatory clearance and clinical adoption, spectral flow cytometry could pave the way for the development of sophisticated, high-dimensional assay kits, facilitating their wider use in clinical diagnostics [105].

### 6.2. Approaches Requiring Further Developments

While fluorescence-based flow cytometry remains the predominant method, it is not the only approach available for cell analysis. Alternative techniques such as surface plasmon resonance (SPR), surface-enhanced Raman scattering (SERS), and electrical impedance provide additional methodologies for expanding the range of detectable analytes and gaining deeper insights into cellular properties beyond fluorescence signals [106]. Surface plasmon resonance (SPR) has been extensively utilized over the past three decades to measure label-free biomolecular interactions in close proximity to the sensor surface (within ~300 nm) in real time. This technique allows for the analysis of cells based on specific properties of interest, with a focus on label-free detection of cellular interactions and molecular binding events. SPR is traditionally capable of detecting interactions involving molecules with molecular weights ranging from 1000 Daltons to 500 kilodaltons. Currently, its primary application is in cell imaging [107].

Surface-enhanced Raman scattering (SERS) leveraging metal nanoparticles can achieve signal intensities on par with fluorescence, but with significantly narrower spectral features, which allows for an enhanced degree of multiplexing. Raman resonant compounds adsorbed onto the nanoparticles confer a unique spectral fingerprint to each SERS tag [108]. These tags are subsequently encapsulated in a polymer coating, enabling conjugation to antibodies or other targeting molecules. The narrow spectral signatures of SERS signals facilitate the detection of a greater number of distinct probes within a confined region of the optical spectrum using a single laser and detector, thereby permitting higher levels of multiplexing and comprehensive multiparameter analysis [109]. Although SERS tags present distinct advantages as labels for antibodies or other ligands, fluorescent dyes offer a broader range of applications that SERS tags cannot match. These applications include pH or ion sensing, enzyme substrates, and viability dyes, among others.

### 6.3. The Emergence of Assistive Analytical Tools

The generation of standardized multiparametric data at the single-cell level should improve the diagnosis of neoplasm. However, the amount and complexity of the data generated can be difficult to handle, even for experienced cytometrists. This is even more true when large numbers of samples are analyzed retrospectively. To support biologists in this endeavor several mathematical-based approaches have been developed. For instance, the visualization of multiple dimensions can be simplified using dimensionality reduction algorithms such as t-distributed stochastic neighbor embedding (t-SNE) and uniform manifold approximation and projection (UMAP) [110]. The classical manual gating strategy approach which is biased by the user’s expertise and experience becomes obsolete when multiple markers are applied and when their expression profile is unknown. Hence, supervised and unsupervised approaches can be used to enhance the outcomes of the analysis. Clustering algorithms such as self-organizing maps (FlowSOM) assist with combining cells into groups and allow cells to be used as separate groups within visualization plots to highlight differences between groups.

Artificial intelligence, specifically machine learning (ML) is increasingly being applied to flow cytometry data in hematological malignancies to improve analysis efficiency and accuracy, offering practical benefits in the clinical laboratory. For instance, ML algorithms can automate auto-gating, the process of identifying and quantifying cell populations, reducing manual effort and variability, as explored in studies on unsupervised pattern recognition methods for flow data [111]. They are also used to aid in the classification of malignancy subtypes by recognizing complex immunophenotypic patterns associated with specific conditions, assisting in differentiating between normal and malignant samples or classifying different leukemia types [112]. Furthermore, ML holds promise for enhancing the sensitivity of MRD detection by training models to identify rare aberrant cells among millions, a challenging task that can be made more robust with advanced analytical approaches as demonstrated in AML [113] and myelodysplastic syndrome diagnosis and prognosis [114]. These applications aim to make flow cytometry analysis faster, more objective, and more powerful for diagnosing and monitoring these diseases.

## 7. Conclusions

Flow cytometry is essential for diagnosing and managing hematological malignancies, providing detailed insights into the cellular composition of blood, bone marrow, and other sample types. The contributions of flow cytometry are as follows: (i) identification of abnormal populations, (ii) characterization of leukemias and lymphomas, (iii) monitoring minimal residual disease, (iv) evaluating treatment response, and (v) guiding personalized medicine. While recent advances in hardware may enable the extension of the number of markers used, improved standardization of the whole process is needed to ensure the quality and reproducibility of the results. In this review, we have covered a non-exhaustive list of gaps in the use of flow cytometry [115]. The variability introduced during sample preparation (pre-analytical phase) can be significant. However, solutions exist to minimize the variability introduced by manual pipetting and ensure better tracking of the whole process. This includes the use of ready-to-use reagents, the appropriate calibration and standardization of the analytical phase, and the use of assistive software for the post-analytical phase [116,117,118]. Whenever this is not possible, other solutions exist, such as the automation for cocktailing liquid antibodies and sample preparation [119]. Whichever is chosen, it should improve the quality of the flow cytometry data generated. Once these issues are minimized, the use of more advanced technologies such as spectral flow cytometry could bring value to diagnostic laboratories.

## Figures and Tables

**Table 1:** Markers generally used for the screening of some hematological malignancies.

AML	B-Cell ALL	T-Cell ALL	CLL	CML ^#^	B-Cell Lymphoma	T-Cell Lymphoma
CD13	CD10	CD1a	CD5	CD11b	CD19	CD2
CD14	CD19	CD2	CD19	CD13	CD20	CD3
CD33	CD20	CD3	CD20	CD14	CD22	CD4
CD34	CD22	CD5	CD23	CD33	CD79a	CD5
CD45	CD34	CD7	CD38	CD34	CD5 ^$,&^	CD7
CD64	CD45	CD45	CD43	CD45	CD10 *	CD8
CD117	TdT	TdT	CD79b		CD23 ^&^	CD30 ^§^
HLA-DR			CD200		CD30 ^§^	
MPO			FMC7		BCL2 *	
			kappa/lambda		Cyclin D1 ^$^	

^#^ the definitive diagnosis of CML is performed with proving the t(9;22) Philadelphia chromosome translocation (by FISH), or BCR-ABL1 fusion gene (qRT-PCR). * follicular lymphoma. ^$^ mantle cell lymphoma. ^&^ CLL/SLL. ^§^ anaplastic large cell lymphoma. TdT: terminal deoxynucleotidyl transferase. MPO: myeloperoxidase. TRBC1 and TRBC2 can be used for the assessment of T cell clonality.

## References

[1] Brown M., Wittwer C. (2000). Flow cytometry: Principles and clinical applications in hematology. Clin. Chem..

[2] Laerum O.D., Farsund T. (1981). Clinical application of flow cytometry: A review. Cytometry.

[3] Cenariu M., Grewal R., Bumbea H., Sauma D., Tomuleasa C. (2023). Editorial: Flow cytometry—A powerful tool for diagnosis and therapy monitoring in hematology and immunology. Front. Med..

[4] Lucas F., Hergott C.B. (2023). Advances in Acute Myeloid Leukemia Classification, Prognostication and Monitoring by Flow Cytometry. Clin. Lab. Med..

[5] Manohar S.M., Shah P., Nair A. (2021). Flow cytometry: Principles, applications and recent advances. Bioanalysis.

[6] Song Y., Lee Y. (2024). Brief guide to flow cytometry. Mol. Cells.

[7] Radcliff G., Jaroszeski M.J. (1998). Basics of flow cytometry. Methods Mol. Biol..

[8] Telford W.G. (2024). Laser Sources for Traditional and Spectral Flow Cytometry. Methods Mol. Biol..

[9] Lagoo A.S. (2023). How to Design and Validate a Clinical Flow Cytometry Assay. Clin. Lab. Med..

[10] Scheuermann R.H., Bui J., Wang H.Y., Qian Y. (2017). Automated Analysis of Clinical Flow Cytometry Data: A Chronic Lymphocytic Leukemia Illustration. Clin. Lab. Med..

[11] Muronova L., Soucek O., Zihala D., Sevcikova T., Popkova T., Plonkova H., Venglar O., Pour L., Stork M., Rihova L. (2025). Real-World Evidence on Prognostic Value of MRD in Multiple Myeloma Using Flow Cytometry. Eur. J. Haematol..

[12] Bain B.J. (2022). Flow Cytometry of Hematological Malignancies. Br. J. Haematol..

[13] Beck J.D., Andreeff M., Mertelsmann R., Haghbin M., Tan C., Miller D.R., Good R.A., Gupta S. (1980). Childhood CML in blastic stage: An analysis of cell markers and cell kinetics. Am. J. Hematol..

[14] Lanier L.L., Warner N.L., Ledbetter J.A., Herzenberg L.A. (1981). Quantitative immunofluorescent analysis of surface phenotypes of murine B cell lymphomas and plasmacytomas with monoclonal antibodies. J. Immunol..

[15] Alatawi D., Bakhsh I., Tashkandi S., Alqarni A., Shaik A.P., Abudawood M. (2023). Diagnostic Accuracy of Flow Cytometric DNA Index in Saudi Children with B Cell Acute Lymphoblastic Leukemia. Children.

[16] Soilleux E.J., Rodgers D.T., Situ J.J., Evans S.C., Konda V.N., Yang H.C., Pang J., Gilbey Smith I., Rajesh P., Salimi M. (2024). Demonstration of T-Cell Monotypia Using Anti-TCRbeta1/2 (TRBC1/2) Immunostaining as a Rapid and Cost-Effective Alternative to PCR-Based Clonality Studies for the Diagnosis of T-Cell Lymphoma. Diagnostics.

[17] Eyre T.A., Bishton M.J., McCulloch R., O’Reilly M., Sanderson R., Menon G., Iyengar S., Lewis D., Lambert J., Linton K.M. (2024). Diagnosis and management of mantle cell lymphoma: A British Society for Haematology Guideline. Br. J. Haematol..

[18] Killick S.B., Wiseman D.H., Quek L., Cargo C., Culligan D., Enright H., Green S., Ingram W., Jones G.L., Kell J. (2021). British Society for Haematology guidelines for the diagnosis and evaluation of prognosis of Adult Myelodysplastic Syndromes. Br. J. Haematol..

[19] Sive J., Cuthill K., Hunter H., Kazmi M., Pratt G., Smith D., British Society of Haematology (2021). Guidelines on the diagnosis, investigation and initial treatment of myeloma: A British Society for Haematology/UK Myeloma Forum Guideline. Br. J. Haematol..

[20] Ng A.P., Adams R., Tiong I.S., Seymour L., Talaulikar D., Palfreyman E., Enjeti A., Tate C. (2024). Reporting bone marrow biopsies for myelodysplastic neoplasms and acute myeloid leukaemia incorporating WHO 5th edition and ICC 2022 classification systems: ALLG/RCPA joint committee consensus recommendations. Pathology.

[21] Debliquis A., Ahle G., Houillier C., Soussain C., Hoang-Xuan K., Le Garff-Tavernier M., CytHem and in partnership with the LOC Network (2024). Analysis of cerebrospinal fluid for the diagnosis of CNS lymphoma: Comparison of the ESCCA/ISCCA protocol and real-world data of the CytHem/LOC French network. Cytom. Part B Clin. Cytom..

[22] Trka J., Fronkova E. (2025). Minimal residual disease detection for acute lymphoblastic leukaemia in peripheral blood-Are we there yet?. Br. J. Haematol..

[23] Khoury J.D., Solary E., Abla O., Akkari Y., Alaggio R., Apperley J.F., Bejar R., Berti E., Busque L., Chan J.K.C. (2022). The 5th edition of the World Health Organization Classification of Haematolymphoid Tumours: Myeloid and Histiocytic/Dendritic Neoplasms. Leukemia.

[24] Westers T.M., Ireland R., Kern W., Alhan C., Balleisen J.S., Bettelheim P., Burbury K., Cullen M., Cutler J.A., Della Porta M.G. (2012). Standardization of flow cytometry in myelodysplastic syndromes: A report from an international consortium and the European LeukemiaNet Working Group. Leukemia.

[25] Rodgers G., Young N.S. (2024). Bethesda Handbook of Clinical Hematology.

[26] Rawstron A.C., Paiva B., Stetler-Stevenson M. (2016). Assessment of minimal residual disease in myeloma and the need for a consensus approach. Cytom. Part B Clin. Cytom..

[27] Marchevsky A.M., Gupta R., Balzer B. (2010). Diagnosis of metastatic neoplasms: A clinicopathologic and morphologic approach. Arch. Pathol. Lab. Med..

[28] Chen X., Fromm J.R., Naresh K.N. (2022). “Blasts” in myeloid neoplasms—How do we define blasts and how do we incorporate them into diagnostic schema moving forward?. Leukemia.

[29] Martig D.S., Fromm J.R. (2022). A comparison and review of the flow cytometric findings in classic Hodgkin lymphoma, nodular lymphocyte predominant Hodgkin lymphoma, T cell/histiocyte rich large B cell lymphoma, and primary mediastinal large B cell lymphoma. Cytom. Part B Clin. Cytom..

[30] Tembhare P., Yuan C.M., Xi L., Morris J.C., Liewehr D., Venzon D., Janik J.E., Raffeld M., Stetler-Stevenson M. (2011). Flow cytometric immunophenotypic assessment of T-cell clonality by Vβ repertoire analysis: Detection of T-cell clonality at diagnosis and monitoring of minimal residual disease following therapy. Am. J. Clin. Pathol..

[31] Chen J., Gale R.P., Hu Y., Yan W., Wang T., Zhang W. (2024). Measurable residual disease (MRD)-testing in haematological and solid cancers. Leukemia.

[32] Seferna K., Svaton M., Rennerova A., Skotnicova A., Reznickova L., Valova T., Sedlacek P., Riha P., Formankova R., Keslova P. (2025). NGS-MRD negativity in post-HSCT ALL spares unnecessary therapeutic interventions triggered by borderline qPCR results without an increase in relapse risk. HemaSphere.

[33] Cherian S., Hedley B.D., Keeney M. (2019). Common flow cytometry pitfalls in diagnostic hematopathology. Cytom. Part B Clin. Cytom..

[34] Song J.Y., Pan Z. (2025). Aberrant expression in lymphoma, a diagnostic pitfall. Hum. Pathol..

[35] Frye Naharro E., Peterson D., Yohe S.L., Linden M.A. (2024). Application and pitfalls of immunophenotyping in challenging plasma cell neoplasms: A case series. Hum. Pathol..

[36] Placek A., Lockhart B., Miller K.P., Wertheim G.B., Maude S.L., Wood B.L., Kovach A.E. (2024). Maturational dyssynchrony in benign B-cell precursors following lymphocyte depleting chemotherapy: A potential pitfall for B-lymphoblastic leukemia minimal/measurable residual disease (MRD) flow cytometry analysis. Cytom. Part B Clin. Cytom..

[37] Soma L., Wu D., Chen X., Edlefsen K., Fromm J.R., Wood B. (2015). Apparent CD19 expression by natural killer cells: A potential confounder for minimal residual disease detection by flow cytometry in B lymphoblastic leukemia. Cytom. Part B Clin. Cytom..

[38] Wood B.L., Levin G.R. (2006). Interactions between mouse IgG2 antibodies are common and mediated by plasma C1q. Cytom. Part B Clin. Cytom..

[39] Golzari-Sorkheh M., Yoganathan K., Chen E.L.Y., Singh J., Zúñiga-Pflücker J.C. (2025). T Cell Development: From T-Lineage Specification to Intrathymic Maturation. Adv. Exp. Med. Biol..

[40] Xing R., Wang M., Wang L., Pan M., Wang Y., Zhou H. (2025). Clinical updates of B-cell maturation antigen-targeted therapy in multiple myeloma (MM) and relapsed/refractory MM (Review). Int. J. Mol. Med..

[41] Costa B.A., Ortiz R.J., Lesokhin A.M., Richter J. (2024). Soluble B-cell maturation antigen in multiple myeloma. Am. J. Hematol..

[42] Weiskopf K., Schnorr P.J., Pang W.W., Chao M.P., Chhabra A., Seita J., Feng M., Weissman I.L. (2016). Myeloid Cell Origins, Differentiation, and Clinical Implications. Microbiol. Spectr..

[43] Vesper H.W., Myers G.L., Miller W.G. (2016). Current practices and challenges in the standardization and harmonization of clinical laboratory tests. Am. J. Clin. Nutr..

[44] Panteghini M., Forest J.C. (2005). Standardization in laboratory medicine: New challenges. Clin. Chim. Acta Int. J. Clin. Chem..

[45] Wears R.L. (2015). Standardisation and Its Discontents. Cogn. Technol. Work.

[46] Rajab A., Axler O., Leung J., Wozniak M., Porwit A. (2017). Ten-color 15-antibody flow cytometry panel for immunophenotyping of lymphocyte population. Int. J. Lab. Hematol..

[47] Chong H.M., Zhang Z.W., Li J.M., Ren X.D., Gong C.M., Zhu Z.X., Xiang N., Ni Z.H., Huang Q. (2025). Cost-effective in-house-made whole blood materials for internal quality control in clinical flow cytometry analysis. Anal. Bioanal. Chem..

[48] Tettero J.M., Al-Badri W.K.W., Ngai L.L., Bachas C., Breems D.A., van Elssen C.H.M.J., Fischer T., Gjertsen B.T., van Gorkom G.N.Y., Gradowska P. (2022). Concordance in measurable residual disease result after first and second induction cycle in acute myeloid leukemia: An outcome- and cost-analysis. Front. Oncol..

[49] Ross A., Rudd D., Wight J. (2025). Low flow: Selecting a limited flow cytometry panel where resources are constrained. Blood Rev..

[50] Valle A., Maugeri N., Manfredi A.A., Battaglia M. (2012). Standardization in flow cytometry: Correct sample handling as a priority. Nat. Rev. Immunol..

[51] Plank K., Dorn C., Krause S.W. (2021). The effect of erythrocyte lysing reagents on enumeration of leukocyte subpopulations compared with a no-lyse-no-wash protocol. Int. J. Lab. Hematol..

[52] Kasinrerk W. (2003). A flow cytometric method for enumeration of lymphocyte sub-populations in sample containing lysis-resistant red blood cells. Immunol. Lett..

[53] Johansson U., Macey M. (2014). Tandem dyes: Stability in cocktails and compensation considerations. Cytom. Part B Clin. Cytom..

[54] Siddiqui S., Livák F. (2023). Principles of Advanced Flow Cytometry: A Practical Guide. Methods Mol. Biol..

[55] Kala P.S., Zubair M. (2024). Flow Cytometry Blood Cell Identification. StatPearls.

[56] Meyerson H., Lazarus H., Schmaier A. (2019). Flow Cytometry in Hematology. Concise Guide to Hematology.

[57] Craig F.E., Foon K.A. (2008). Flow cytometric immunophenotyping for hematologic neoplasms. Blood.

[58] Gorczyca W. (2022). Flow Cytometry in Neoplastic Hematology. Morphologic-Immunophenotypic-Genetic Correlation.

[59] Wang L., Hoffman R.A. (2017). Standardization, Calibration, and Control in Flow Cytometry. Curr. Protoc. Cytom..

[60] Gonneau C., Wang L., Mitra-Kaushik S., Trampont P.C., Litwin V. (2021). Progress towards global standardization for quantitative flow cytometry. Bioanalysis.

[61] Kalina T. (2020). Reproducibility of Flow Cytometry Through Standardization: Opportunities and Challenges. Cytom. Part A J. Int. Soc. Anal. Cytol..

[62] Piccoli S., Mehta D., Vitaliti A., Allinson J., Amur S., Eck S., Green C., Hedrick M., Hopper S., Ji A. (2019). 2019 White Paper on Recent Issues in Bioanalysis: FDA Immunogenicity Guidance, Gene Therapy, Critical Reagents, Biomarkers and Flow Cytometry Validation (Part 3—Recommendations on 2019 FDA Immunogenicity Guidance, Gene Therapy Bioanalytical Challenges, Strategies for Critical Reagent Management, Biomarker Assay Validation, Flow Cytometry Validation & CLSI H62). Bioanalysis.

[63] Stelzer G.T., Marti G., Hurley A., McCoy P., Jr Lovett E.J., Schwartz A. (1997). U.S.-Canadian Consensus recommendations on the immunophenotypic analysis of hematologic neoplasia by flow cytometry: Standardization and validation of laboratory procedures. Cytometry.

[64] ICSH/ICCS Workgroup (2013). Validation of cell-based fluorescence assays: Practice guidelines from the International Council for Standardization of Haematology and International Clinical Cytometry Society. Cytom. Part B Clin. Cytom..

[65] https://www.cytometry.org/web/quality.php.

[66] Le Lann L., Jouve P.E., Alarcón-Riquelme M., Jamin C., Pers J.O., PRECISESADS Flow Cytometry Study Group, PRECISESADS Clinical Consortium (2020). Standardization procedure for flow cytometry data harmonization in prospective multicenter studies. Sci. Rep..

[67] Porwit A., Béné M.C., Duetz C., Matarraz S., Oelschlaegel U., Westers T.M., Wagner-Ballon O., Kordasti S., Valent P., Preijers F. (2023). Multiparameter flow cytometry in the evaluation of myelodysplasia: Analytical issues: Recommendations from the European LeukemiaNet/International Myelodysplastic Syndrome Flow Cytometry Working Group. Cytometry. Part B Clin. Cytom..

[68] Keeney M., Barnett D., Gratama J.W. (2004). Impact of standardization on clinical cell analysis by flow cytometry. J. Biol. Regul. Homeost. Agents.

[69] Monaghan S.A., Eck S., Bunting S., Dong X.X., Durso R.J., Gonneau C., Hays A., Illingworth A., League S.C., Linskens E. (2024). Flow cytometry assay modifications: Recommendations for method validation based on CLSI H62 guidelines. Cytom. Part B Clin. Cytom..

[70] Westera L., van Viegen T., Jeyarajah J., Azad A., Bilsborough J., van den Brink G.R., Cremer J., Danese S., D’Haens G., Eckmann L. (2017). Centrally Determined Standardization of Flow Cytometry Methods Reduces Interlaboratory Variation in a Prospective Multicenter Study. Clin. Transl. Gastroenterol..

[71] Glier H., Heijnen I., Hauwel M., Dirks J., Quarroz S., Lehmann T., Rovo A., Arn K., Matthes T., Hogan C. (2019). Standardization of 8-color flow cytometry across different flow cytometer instruments: A feasibility study in clinical laboratories in Switzerland. J. Immunol. Methods.

[72] Debliquis A., Baseggio L., Bouyer S., Guy J., Garnache-Ottou F., Genevieve F., Mayeur-Rousse C., Letestu R., Chapuis N., Harrivel V. (2021). Multicentric MFI30 study: Standardization of flow cytometry analysis of CD30 expression in non-Hodgkin lymphoma. Cytom. Part B Clin. Cytom..

[73] Soh K.T., Wallace P.K. (2021). Evaluation of measurable residual disease in multiple myeloma by multiparametric flow cytometry: Current paradigm, guidelines, and future applications. Int. J. Lab. Hematol..

[74] Schuurhuis G.J., Ossenkoppele G.J., Kelder A., Cloos J. (2018). Measurable residual disease in acute myeloid leukemia using flow cytometry: Approaches for harmonization/standardization. Expert Rev. Hematol..

[75] Ikoma-Colturato M.R.V., Bertolucci C.M., Conti-Spilari J.E., Oliveira E., Simioni A.J., Figueredo-Pontes L.L., Furtado F.M., Alegretti A.P., Azambuja A.P., Gevert F. (2023). Multicentric standardization of minimal/measurable residual disease in B-cell precursor acute lymphoblastic leukaemia using next-generation flow cytometry in a low/middle-level income country. Br. J. Haematol..

[76] Sommer U., Eck S., Marszalek L., Stewart J.J., Bradford J., McCloskey T.W., Green C., Vitaliti A., Oldaker T., Litwin V. (2021). High-sensitivity flow cytometric assays: Considerations for design control and analytical validation for identification of Rare events. Cytom. Part B Clin. Cytom..

[77] Rawstron A.C., Kreuzer K.A., Soosapilla A., Spacek M., Stehlikova O., Gambell P., McIver-Brown N., Villamor N., Psarra K., Arroz M. (2018). Reproducible diagnosis of chronic lymphocytic leukemia by flow cytometry: An European Research Initiative on CLL (ERIC) & European Society for Clinical Cell Analysis (ESCCA) Harmonisation project. Cytom. Part B Clin. Cytom..

[78] Engelmann R., Flores-Montero J., Schilperoord-Vermeulen J., Ritgen M., Hengeveld P.J., Kohlscheen S., Grigore G., Rodriguez R.F., Lecrevisse Q., Philippé J. (2025). Novel Flow Cytometric Antibody Panel and Dedicated Analysis Algorithm for Automated Fully Standardized Minimal Residual Disease Detection in Chronic Lymphocytic Leukemia. Am. J. Hematol..

[79] Terra R., Éthier V., Busque L., Morin-Quintal A., D’Angelo G., Hébert J., Wang X., Lépine G., LeBlanc R., Bergeron J. (2025). Improved identification of clinically relevant Acute Leukemia subtypes using standardized EuroFlow panels versus non-standardized approach. Cytom. Part B Clin. Cytom..

[80] Verbeek M.W.C., van der Velden V.H.J. (2024). The Evolving Landscape of Flowcytometric Minimal Residual Disease Monitoring in B-Cell Precursor Acute Lymphoblastic Leukemia. Int. J. Mol. Sci..

[81] Matarraz S., Leoz P., Yeguas-Bermejo A., van der Velden V., Bras A.E., Sánchez Gallego J.I., Lecrevisse Q., Ayala-Bueno R., Teodosio C., Criado I. (2023). Baseline immunophenotypic profile of bone marrow leukemia cells in acute myeloid leukemia with nucleophosmin-1 gene mutation: A EuroFlow study. Blood Cancer J..

[82] https://euroflow.org.

[83] Raskovalova T., Scheffen L., Jacob M.C., Chevalier S., Tondeur S., Bulabois B., Meunier M., Szymanski G., Lefebvre C., Planta C. (2022). Flow cytometry lyophilised-reagent tube for quantifying peripheral blood neutrophil myeloperoxidase expression in myelodysplastic syndromes (MPO-MDS-Develop): Protocol for a diagnostic accuracy study. BMJ Open.

[84] Hedley B.D., Cheng G., Luider J., Kern W., Lozanski G., Chin-Yee I., Lowes L.E., Keeney M., Careaga D., Magari R. (2018). Initial flow cytometric evaluation of the Clearllab lymphoid screen. Cytom. Part B Clin. Cytom..

[85] Hedley B.D., Cheng G., Keeney M., Kern W., Padurean A., Luider J., Chin-Yee I., Lowes L.E., Rohrbach J., Ortega R. (2021). A multicenter study evaluation of the ClearLLab 10C panels. Cytom. Part B Clin. Cytom..

[86] Smallwood C., Galama L., Apoll L., Heinrich K., Buchanan S., Demers J. (2015). Examining the economic impact of laboratory developed testing in flow cytometry immunophenotyping for hematologic malignancies: An analysis of health resource utilization. Value Health.

[87] George G.V., Kajstura M., Evans A.G., Syposs C.R. (2024). Gamma-delta T-cell acute lymphoblastic lymphoma/leukemia: A report of a rare entity. J. Hematop..

[88] Glencross D.K., Swart L., Pretorius M., Lawrie D. (2022). Evaluation of fixed-panel, multicolour ClearLLab 10C at an academic flow cytometry laboratory in Johannesburg, South Africa. Afr. J. Lab. Med..

[89] Espasa A., Torrents S., Morales-Indiano C., Rico L.G., Bardina J., Ancochea A., Bistué-Rovira À., Linio R., Raya M., Vergara S. (2021). Diagnostic performance of the ClearLLab 10C B cell tube. Cytom. Part B Clin. Cytom..

[90] Streitz M., Miloud T., Kapinsky M., Reed M.R., Magari R., Geissler E.K., Hutchinson J.A., Vogt K., Schlickeiser S., Kverneland A.H. (2013). Standardization of whole blood immune phenotype monitoring for clinical trials: Panels and methods from the ONE study. Transplant. Res..

[91] Maecker H.T., McCoy J.P., Amos M., Elliott J., Gaigalas A., Wang L., Aranda R., Banchereau J., Boshoff C., the FOCIS Human Immunophenotyping Consortium (2010). A model for harmonizing flow cytometry in clinical trials. Nat. Immunol..

[92] Sawitzki B., Harden P.N., Reinke P., Moreau A., Hutchinson J.A., Game D.S., Tang Q., Guinan E.C., Battaglia M., Burlingham W.J. (2020). Regulatory cell therapy in kidney transplantation (The ONE Study): A harmonised design and analysis of seven non-randomised, single-arm, phase 1/2A trials. Lancet.

[93] Kajstura M., LaBarge T., Evans A.G. (2025). ClearLLab 10C reagents panel can be applied to analyze paucicellular samples by flow cytometry. Cytom. Part B Clin. Cytom..

[94] Frater J.L., Shirai C.L., Brestoff J.R. (2022). Technological features of blast identification in the cerebrospinal fluid: A systematic review of flow cytometry and laboratory haematology methods. Int. J. Lab. Hematol..

[95] Bouriche L., Bernot D., Nivaggioni V., Arnoux I., Loosveld M. (2019). Detection of Minimal Residual Disease in B Cell Acute Lymphoblastic Leukemia Using an Eight-Color Tube with Dried Antibody Reagents. Cytom. Part B Clin. Cytom..

[96] Ho C., Syed M., Roshal M., Petrova-Drus K., Moung C., Yao J., Quesada A.E., Benhamida J., Vanderbilt C., Liu Y. (2021). Routine Evaluation of Minimal Residual Disease in Myeloma Using Next-Generation Sequencing Clonality Testing: Feasibility, Challenges, and Direct Comparison with High-Sensitivity Flow Cytometry. J. Mol. Diagn. JMD.

[97] Royston D.J., Gao Q., Nguyen N., Maslak P., Dogan A., Roshal M. (2016). Single-Tube 10-Fluorochrome Analysis for Efficient Flow Cytometric Evaluation of Minimal Residual Disease in Plasma Cell Myeloma. Am. J. Clin. Pathol..

[98] Nilsson M., Malmgren H., Samiotaki M., Kwiatkowski M., Chowdhary B.P., Landegren U. (1994). Padlock probes: Circularizing oligonucleotides for localized DNA detection. Science.

[99] Larsson C., Koch J., Nygren A., Janssen G., Raap A.K., Landegren U., Nilsson M. (2004). In situ genotyping individual DNA molecules by target-primed rolling-circle amplification of padlock probes. Nat. Methods.

[100] Chen L., Eriksson A., Weström S., Pandzic T., Lehmann S., Cavelier L., Landegren U. (2022). Ultra-sensitive monitoring of leukemia patients using superRCA mutation detection assays. Nat. Commun..

[101] Robinson J.P., Ostafe R., Iyengar S.N., Rajwa B., Fischer R. (2023). Flow Cytometry: The Next Revolution. Cells.

[102] Brestoff J.R. (2023). Full spectrum flow cytometry in the clinical laboratory. Int. J. Lab. Hematol..

[103] Duffield A.S., Aoki J., Levis M., Cowan K., Gocke C.D., Burns K.H., Borowitz M.J., Vuica-Ross M. (2012). Clinical and pathologic features of secondary acute promyelocytic leukemia. Am. J. Clin. Pathol..

[104] Hayden P.J., O’Connell N.M., O’Brien D.A., O’Rourke P., Lawlor E., Browne P.V. (2006). The value of autofluorescence as a diagnostic feature of acute promyelocytic leukemia. Haematologica.

[105] Zhang T., Gao M., Chen X., Gao C., Feng S., Chen D., Wang J., Zhao X., Chen J. (2022). Demands and technical developments of clinical flow cytometry with emphasis in quantitative, spectral, and imaging capabilities. Nanotechnol. Precis. Eng..

[106] Watson D.A., Brown L.O., Gaskill D.F., Naivar M., Graves S.W., Doorn S.K., Nolan J.P. (2008). A flow cytometer for the measurement of Raman spectra. Cytometry. Part A J. Int. Soc. Anal. Cytol..

[107] Xu J., Zhang P., Chen Y. (2024). Surface Plasmon Resonance Biosensors: A Review of Molecular Imaging with High Spatial Resolution. Biosensors.

[108] Nolan J.P., Duggan E., Liu E., Condello D., Dave I., Stoner S.A. (2012). Single cell analysis using surface enhanced Raman scattering (SERS) tags. Methods.

[109] Guo H., Zhi S., Zhao Z., Gao T., Ma H., Luo S.H., Zhang W., Guo P., Ren B., Tian Z.Q. (2025). Rapid SERS Analysis: From Laboratory to Real Sample. ACS Appl. Mater. Interfaces.

[110] Spies N.C., Rangel A., English P., Morrison M., O’Fallon B., Ng D.P. (2025). Machine Learning Methods in Clinical Flow Cytometry. Cancers.

[111] Seheult J.N., Otteson G.E., Weybright M.J., Timm M.M., Han W., Jevremovic D., Horna P., Olteanu H., Shi M. (2025). Clinical Validation and Post-Implementation Performance Monitoring of a Neural Network-Assisted Approach for Detecting Chronic Lymphocytic Leukemia Minimal Residual Disease by Flow Cytometry. Cancers.

[112] Pedreira C.E., Sobral da Costa E., Lecrevise Q., Grigore G., Fluxa R., Verde J., Hernandez J., van Dongen J.J.M., Orfao A. (2019). From big flow cytometry datasets to smart diagnostic strategies: The EuroFlow approach. J. Immunol. Methods.

[113] Mocking T.R., van de Loosdrecht A.A., Cloos J., Bachas C. (2025). Applications of machine learning for immunophenotypic measurable residual disease assessment has proven its utility in acute myeloid leukemia. HemaSphere.

[114] Stagno F., Mirabile G., Rizzotti P., Bottaro A., Pagana A., Gangemi S., Allegra A. (2025). Using Artificial Intelligence to Enhance Myelodysplastic Syndrome Diagnosis, Prognosis, and Treatment. Biomedicines.

[115] Czechowska K., Lannigan J., Aghaeepour N., Back J.B., Begum J., Behbehani G., Bispo C., Bitoun D., Fernández A.B., Boova S.T. (2019). Cyt-Geist: Current and Future Challenges in Cytometry: Reports of the CYTO 2019 Conference Workshops. Cytometry. Part A J. Int. Soc. Anal. Cytol..

[116] Gorrese M., Bertolini A., Fresolone L., Campana A., Pezzullo L., Guariglia R., Mettivier L., Manzo P., Cuffa B., D’Alto F. (2022). Inter-intra instrument comparison and standardization of a 10-color immunophenotyping for B and T cell non-Hodgkin lymphoma diagnosis and monitoring. J. Immunol. Methods.

[117] Ng D.P., Zuromski L.M. (2021). Augmented Human Intelligence and Automated Diagnosis in Flow Cytometry for Hematologic Malignancies. Am. J. Clin. Pathol..

[118] Shopsowitz K., Lofroth J., Chan G., Kim J., Rana M., Brinkman R., Weng A., Medvedev N., Wang X. (2024). MAGIC-DR: An interpretable machine-learning guided approach for acute myeloid leukemia measurable residual disease analysis. Part B Clin. Cytom..

[119] Al-Attar A., Kumar K.R., Al-Attar A., Untersee D., O’Driscoll M., Ventura M.F.S., Lin L. (2024). Automation in flow cytometry: Guidelines and review of systems. Cytom. Part B Clin. Cytom..

